# Molecular mechanisms governing the progression of nephritis in lupus prone mice and human lupus patients

**DOI:** 10.3389/fimmu.2023.1147526

**Published:** 2023-03-01

**Authors:** Andrea R. Daamen, Hongyang Wang, Prathyusha Bachali, Nan Shen, Kathryn M. Kingsmore, Robert D. Robl, Amrie C. Grammer, Shu Man Fu, Peter E. Lipsky

**Affiliations:** ^1^ AMPEL BioSolutions LLC, Charlottesville, VA, United States; ^2^ Center for Immunity, Inflammation, and Regenerative Medicine, Department of Medicine, University of Virginia School of Medicine, Charlottesville, VA, United States; ^3^ Division of Rheumatology, Department of Medicine, University of Virginia School of Medicine, Charlottesville, VA, United States; ^4^ Shanghai Institute of Rheumatology, Renji Hospital, Shanghai Jiao Tong University School of Medicine, Shanghai, China

**Keywords:** lupus, nephritis, transcriptomics, bioinformatics, translational, mouse models

## Abstract

**Introduction:**

Pathologic inflammation is a major driver of kidney damage in lupus nephritis (LN), but the immune mechanisms of disease progression and risk factors for end organ damage are poorly understood.

**Methods:**

To characterize molecular profiles through the development of LN, we carried out gene expression analysis of microdissected kidneys from lupus-prone NZM2328 mice. We examined male mice and the congenic NZM2328.R27 strain as a means to define mechanisms associated with resistance to chronic nephritis. Gene expression profiles in lupus mice were compared with those in human LN.

**Results:**

NZM2328 mice exhibited progress from acute to transitional and then to chronic glomerulonephritis (GN). Each stage manifested a unique molecular profile. Neither male mice nor R27 mice progressed past the acute GN stage, with the former exhibiting minimal immune infiltration and the latter enrichment of immunoregulatory gene signatures in conjunction with robust kidney tubule cell profiles indicative of resistance to cellular damage. The gene expression profiles of human LN were similar to those noted in the NZM2328 mouse suggesting comparable stages of LN progression.

**Conclusions:**

Overall, this work provides a comprehensive examination of the immune processes involved in progression of murine LN and thus contributes to our understanding of the risk factors for end-stage renal disease. In addition, this work presents a foundation for improved classification of LN and illustrates the applicability of murine models to identify the stages of human disease.

## Introduction

Systemic lupus erythematosus (SLE) is an autoimmune disorder that can affect a variety of tissues, including the kidney (1). Lupus nephritis (LN) affects approximately 40% of adult lupus patients with 10-20% of patients developing end-stage renal disease (ESRD) (2). Disease is thought to initiate in the kidney glomerulus with immune complex (IC) deposition and complement activation leading to the release of damage associated molecular patterns (DAMPS), cytokine production, and the infiltration of inflammatory cells that amplify and sustain inflammation (3, 4). Damage to the kidney glomerulus promotes ischemic damage and chronic hypoxia, compromising the downstream blood supply to the tubulointerstitium (TI) and reducing tubule cell viability, which serve as prognostic markers for the development of ESRD (5–7). Despite advances in understanding of LN, there remains no clear indication of factors controlling the conversion of acute to chronic nephritis and no proven treatments to prevent ESRD (8–10).

Previous studies established the NZM2328 lupus-prone mouse strain as a model of human LN, with severe IC-mediated nephritis and early mortality predominantly affecting female mice (11–13). These studies determined that disease in female NZM2328 mice presents in two stages termed acute glomerulonephritis (AGN) with pathology largely confined to the glomerulus, and chronic GN (CGN) in which inflammation and tissue damage are also found amongst and between the tubules (12, 13). AGN and CGN were associated with a genetic loci on chromosome 1, the *Agnz1* and *Cgnz1* regions respectively. In addition, the NZM2328.Lc1R27 (R27) recombinant strain was generated by replacing the *Cgnz1* region of NZM2328 with that from the C57BL/J strain, such that female R27 mice develop AGN but do not progress to CGN. Similarly, male NZM2328 mice develop a milder, acute form of nephritis but do not exhibit severe proteinuria or progress to chronic disease (14, 15).

The heterogeneity in disease presentation among LN patients and difficulty in predicting therapeutic responses have highlighted the utility of molecular profiling to improve classification of lupus kidney pathology (10, 16). Here, to understand the pathogenesis of LN and especially the relationship between acute and chronic disease in greater detail, we utilized transcriptome analysis to define the stages of GN in NZM2328 mice and identify pathologic immune populations and processes associated with disease progression. In addition, we identified distinct mechanisms of resistance to chronic disease based on differences in gender and genetics and demonstrated similarities in gene expression profiles between human and murine LN, suggesting comparable progression with implications for elucidating risk factors for development of ESRD in human lupus patients.

## Materials and methods

### Mice

NZM2328 and NZM2328.R27 congenic mice were obtained/generated as previously described (11, 13). All mice were housed at the University of Virginia (UVA) Center of Comparative Medicine under pathogen-free conditions.

### Histological characterization

Kidneys of NZM2328 and R27 mice were harvested and the stage of GN was confirmed by histological classification (**Supplementary Table 1**) as previously described (11, 13). Briefly, mice were sacrificed at 8-9 weeks for the control group and 26-38 weeks for diseased mice. Before sacrifice, the presence of nephritis in diseased mice was assayed using proteinuria test strips. Mice were classified into AGN, TGN, or CGN stages by assessment of glomerular size and cellularity, mesangial expansion, glomerular sclerosis, kidney fibrosis, tubular cell dilation, tubular atrophy, and immune cell infiltration. IgG, C3, and ANA staining were performed as previously described.

### Laser microdissection and microarray hybridization

LMD of snap frozen kidney sections was performed as previously described (17). Frozen sections were cut by Cryostat to 5-micron thickness and placed on dry ice. The sections were fixed in 70% ethanol followed by hematoxylin and eosin (H&E) staining. LMD was performed along and including the Bowman’s capsule to isolate kidney glomeruli while tubulointerstitial tissue was collected from approximately 3-4 layers of cells outside of the microdissected glomeruli. For each mouse, 40 glomeruli/tissue picks were collected and pooled to prepare each RNA sample.

Total RNA was isolated from LMD-derived cells using PicoPure RNA isolation kit (Applied Biosystems). RNA quality was detected by Agilent Pico Gel. Array hybridization was carried out by the UT Southwestern Microarray Core facility for the Affymetrix Clariom D Array of NZM2328 female and R27 mice and by the UVA Genome Analysis and Technology Core for the GeneChip Mouse 430 v2.0 array of NZM2328 female and male mice according to standard Affymetrix protocols.

### Microarray data processing

Raw CEL files from the publicly available murine IFNα-NZB (GSE86423) and human microdissected kidney (GSE32591) microarray datasets were derived from GEO using the R/Bioconductor package GEOquery. Processing of raw microarray data from all murine and human microarray datasets was carried out with the R/Bioconductor packages oligo, affy, affycoretools, and simpleaffy. Affymetrix CEL files were background corrected and normalized using the Robust Multiarray Average (RMA) or GeneChip Robust Multiarray Average (GCRMA) methods depending on the microarray platform. Normalized data was transformed into log_2_ intensity values and formatted as R expression set objects (E-sets). Principal component analysis (PCA) was used to inspect the datasets for outliers. E-sets were annotated using chip definition files (CDFs) corresponding to Affymetrix Clariom D (NZM2328 female and R27 mice), Mouse 430 v2.0 (NZM2328 male mice), HT_MG-430_PM (IFNα-NZB mice), HGU133A_CDF_ENTREZG_10 (human microdissected kidney), or HG-U133_Plus_2 (human whole kidney) arrays. Low intensity probes were filtered by visual selection of thresholds at the dip in histograms of binned log_2_-transformed probe intensities. Variance correction was carried out using the ebayes function in the R/Bioconductor LIMMA package. Resulting p-values were adjusted for multiple comparisons using the Benjamini-Hochberg correction that produced a false discovery rate (FDR) for each comparison. Probes were distilled down to differentially expressed (DE) probes with FDR < 0.2 which were considered statistically significant.

### Gene set variation analysis (GSVA)

The R/Bioconductor package GSVA (18) (v1.36.3) was used as a non-parametric, unsupervised method to estimate the variation in enrichment of pre-defined gene sets in microarray data from NZM2328 mice. The input for GSVA was a matrix of log_2_ expression values for all samples and a collection of gene signatures for immune cell types and functional pathways. Genes with multiple Affymetrix identifiers were selected based on the highest interquartile range (IQR) and probes with IQR=0 were filtered out. GSVA enrichment scores were calculated on a per sample basis, without specifying the sample labels, using a Kolgomorov-Smirnoff (KS)-like random walk statistic comparing the distribution of genes in the specific gene modules to those not in the module and were scaled across all samples to values between -1 and +1 indicative of negative enrichment and positive enrichment, respectively.

### GSVA gene set generation

Gene sets used as input for GSVA are listed in **Supplementary Table 2**. Cell type and pathway gene signatures were generated based on literature mining, Mouse Genome Informatics (MGI) (19) gene ontology (GO) terms, and immune cell-specific expression derived from the Immunological Genome Project Consortium (ImmGen) (20). The glycolysis, oxidative phosphorylation, amino acid metabolism, and fatty acid oxidation gene signatures have been previously described (21). The cell type gene signatures were derived from Mouse CellScan, a tool for identification of cellular origin from mouse gene expression datasets. The pathway gene signatures were derived from the Mouse Biologically Informed Gene Clustering (BIG-C) tool for categorization of biological functions in mouse gene expression datasets.

### Linear regression analysis

Linear regression analysis between GSVA enrichment scores and log_2_ gene expression values was carried out using GraphPad Prism software (v9.3.1). The goodness of fit is displayed as the R^2^ value. The *p*-value indicates the significance of the slope of the regression line.

### Ingenuity pathway analysis (IPA)

Molecules upstream of selected *Cgnz1* locus genes were identified using IPA upstream regulator (UPR) analysis (Qiagen) (22). UPRs with an overlap p-value < 0.01 were considered significant.

### Multiscale embedded gene co-expression network analysis (MEGENA)

The MEGENA R package (23) was used to generate gene co-expression networks for NZM2328 mouse glomerulus and TI by inputting the top 5,000 row variance genes from the respective gene expression matrices. A planar filtered network (PFN) was formed using a false discovery rate (FDR) of 0.2. MEGENA multi-scale clustering analysis (MCA) used the PFN to form lineages of gene modules which were assigned “lineage” names based on their descendance from the root MEGENA module. Modules were functionally annotated by overlapping their gene symbols with curated mouse-specific functional signatures, immune cell, and kidney tissue cell signatures as well as the top GO terms (24) exhibiting the greatest coverage for each module. Annotations of MEGENA modules were considered significant if there were at least 3 overlapping gene symbols between the module gene symbols and annotation signature gene symbols, and the Fisher’s p-value statistic of the overlap was p<0.2. A module eigengene (ME) was calculated for each module equivalent to the first principal component of a module’s gene expression. Intracorrelations of sample traits were calculated for brief inspection. MEs were correlated to all sample traits and correlations were zeroed out where the p-value of the correlation was >=0.2. All second generation (gen2) MEGENA modules were retained for ensuing analysis. A gene expression set from human whole kidney biopsies was subjected to MEGENA analysis in a similar manner. Gen2 MEGENA modules from NZM2328 glomerulus and TI were examined for preservation in the MEGENA human kidney modules by utilizing an algorithm that generates z.summ composite scores of 20 preservation metrics (25).

### K-means clustering

GSVA enrichment scores of gen2 MEGENA modules (**Supplementary Table 4**) or 22 curated immune cell, kidney cell, and metabolic pathway gene signatures (**Supplementary Table 2**) were used as input for k-means clustering performed with 1000 iterations to identify the most stable clusters for each dataset. Clustering results were visualized using the R package ComplexHeatmap (v 2.12) (26).

### Statistical analysis

P-values and odds ratios (ORs) for the overlap of DEGs with inflammatory cell types and pathways were calculated with a two-sided fisher’s exact test in R with a confidence level of 0.95. All other statistical tests were carried out with GraphPad Prism (v9.3.1). Comparisons for two groups (CTL, AGN) were calculated using an unpaired, two-sided Welch’s t-test. Comparisons for more than two groups (CTL, AGN, TGN, CGN) were calculated using Brown Forsythe and Welch’s ANOVA followed by Dunnett’s T3 multiple comparisons test.

### Study approval

Mice were kept at the University of Virginia Center of Comparative Medicine. All experimental protocols were approved by the Institutional Animal Care and Use committee.

### Data availability

The murine microarray dataset generated for the current study is available from NCBI’s GEO database under accession GSE206806. The human microarray dataset generated for the current study has been submitted to ArrayExpress with accession E-MTAB-12257. The publicly available murine and human microarray datasets analyzed in the current study can be found under GEO accessions GSE86423 and GSE32591, respectively.

## Results

### Renal disease of NZM2328 mice is characterized by escalating stages of inflammation

**Figure 1:**
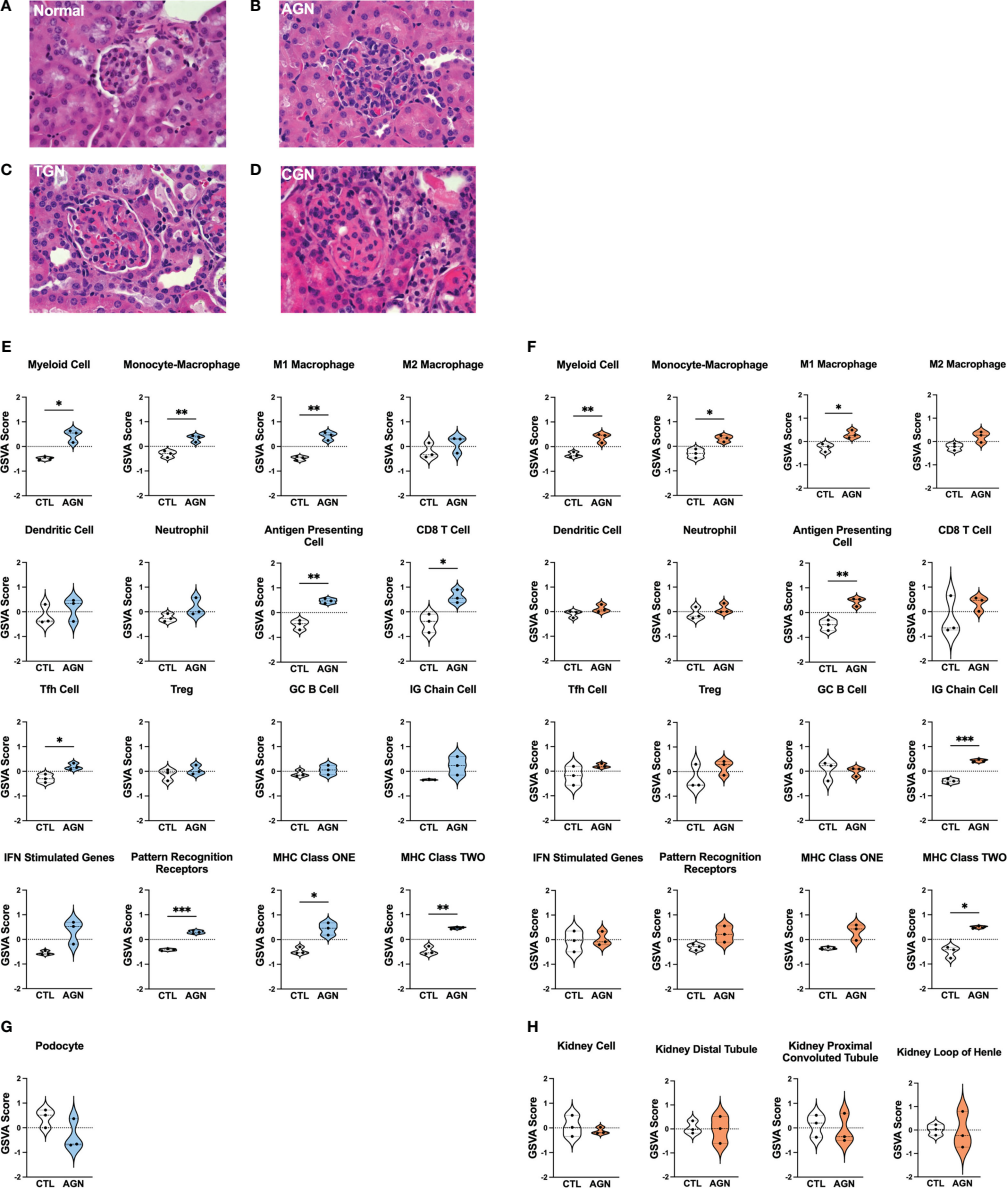
Histologic and transcriptional analysis of immune populations in NZM2328 mice with GN. **(A-D)** H&E staining of kidneys from normal/CTL **(A)** NZM2328 females and mice with acute **(B)**, transitional **(C)**, and chronic **(D)** stage GN. Individual sample gene expression from glomeruli **(E, G)** and TI **(F, H)** of CTL and AGN mice was analyzed by GSVA for enrichment of immune cells/inflammatory pathways **(E, F)** and kidney tissue cells **(G, H)**. Enrichment scores are shown as violin plots. *p<0.05, **p<0.01, ***p<0.001.

To identify different stages of GN in the kidneys of female NZM2328 mice, we carried out histological studies at regular intervals throughout disease progression (**Figures 1A–D**; **Supplementary Table 1**). Tissues from young mice, before disease development and without evidence of kidney pathology were used as a control (**Figure 1A**). At the AGN stage, glomeruli were increased in size with evidence of immune cell infiltration and immune complex deposition including IgG, C3, and anti-nuclear antibody (ANA) deposits (**Figure 1B**). There were no changes to tubule cells of AGN mice and they exhibited mild immune cell infiltration in the interstitium. We identified an additional intermediate stage of disease progression not previously reported termed transitional GN (TGN) at which, like the AGN stage, glomeruli exhibited immune cell infiltration, but levels of IgG and C3 deposition as well as serum levels of anti-DNA antibodies were elevated over AGN mice (**Figure 1C**). The interstitium of TGN mice had more inflammatory cells than at the AGN stage and tubular cells showed some dilation and atrophy. However, tubule damage was not evident histologically. At the CGN stage, mice exhibited glomerular sclerosis, fibrosis with interstitial inflammation, and the highest level of immune complex deposition as compared to earlier disease stages (**Figure 1D**). In CGN stage mice, >80% of tubular cells had tubular dilation with increased evidence of atrophy and tubular casts as compared to the TGN stage.

### Transcriptional profiling uncovers immune populations present at the onset of GN in NZM2328 mice

To establish the inflammatory environment in the kidney at disease onset, we analyzed the transcriptomes of microdissected glomeruli and TI from the kidneys of female NZM2328 mice. Tissues from 8-9 week-old (CTL) mice were used as a control (11–13). Using Gene Set Variation Analysis (GSVA) (18) with a battery of curated gene sets (**Supplementary Table 2**), glomeruli of histologically-defined AGN mice were found to be enriched for gene signatures of a number of immune/inflammatory cell types, including myeloid cells, M1 macrophages (Mϕs), antigen presenting cells (APCs), CD8 T cells, and T follicular helper (Tfh) cells (**Figure 1E**). In addition, genes encoding immune cell receptors, including pattern recognition receptors (PRRs) as well as major histocompatibility complex (MHC) class I and II were significantly elevated in AGN glomeruli. The TI of AGN mice was enriched for many of the same immune signatures, including myeloid cells, M1 Mϕs, APCs, and MHC class II as well as the IG chain signature indicative of the presence of a plasma cell (PC) infiltrate (**Figure 1F**). However, despite the presence of signatures indicative of immune cells, gene signatures of podocytes in the glomeruli (**Figure 1G**) and of tubule cells in the TI (**Figure 1H**) were not significantly different than CTL. Thus, the kidneys of mice with AGN are enriched for predominantly innate immune cell gene signatures with no evidence of damage to the kidney cells.

### Transcriptomic analysis reveals distinct immune profiles of acute, transitional, and chronic GN in NZM2328 mice

**Figure 2:**
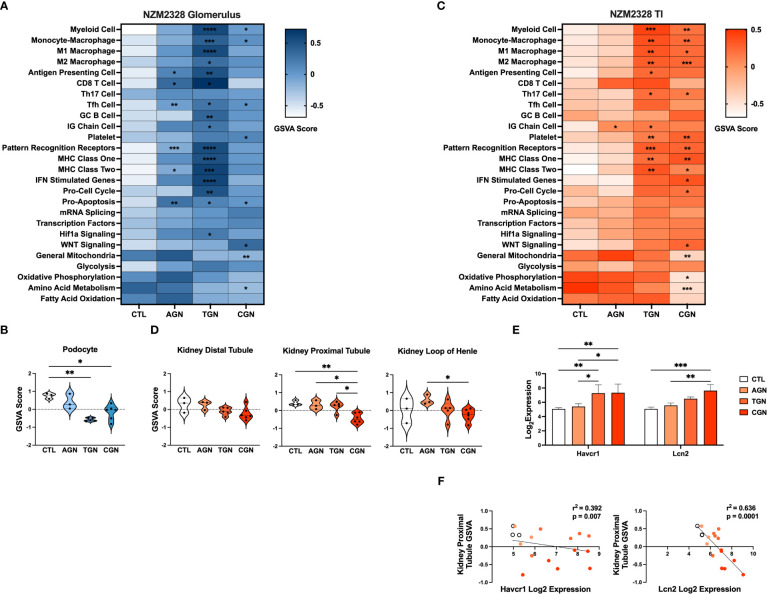
Transcriptomic analysis of GN disease stages in glomeruli and TI of NZM2328 mice. **(A)** Heatmap of GSVA scores for enrichment of immune cell and pathway gene signatures in glomeruli of CTL, AGN, TGN, and CGN mice. Asterisks indicate significant comparisons with CTL mice. **(B)** GSVA enrichment of podocytes in cohorts shown in **(A)**. **(C)** Heatmap of GSVA scores in the TI of cohorts shown in **(A)**. **(D)** GSVA enrichment of kidney tubule cell gene signatures in cohorts from **(C)**. **(E)** Log_2_ expression values of kidney tubule damage-associated genes for cohorts from **(C)**. **(F)** Linear regression between log_2_ expression of kidney tubule damage genes and GSVA scores of kidney tubule cells. *p<0.05, **p<0.01, ***p<0.001, ****p<0.000.

Next, we compared the transcriptomes of glomeruli and TI from pre-disease CTL mice to mice with progressively more severe stages of disease. Overall, we found that the more robust gene signature enrichment in later stages of disease decreased the significant differences between AGN and CTL mice (**Figures 2A, C**). The immune profile of glomeruli of TGN mice reflected an intermediate stage of renal disease and the peak of inflammatory signature enrichment. We found enrichment of gene signatures of germinal center (GC) B cells, myeloid cells, and Mϕs, including both M1 and M2 subsets, as well as signatures of interferon (IFN) stimulated genes, MHC class I, the cell cycle, and the Hif1a signaling pathway (**Figure 2A**). In addition, the inflammatory signatures enriched in AGN mice were increased further at the transitional stage. Glomeruli of CGN mice were enriched for platelets and WNT signaling and de-enriched for gene signatures of mitochondrial function and amino acid metabolism (**Figure 2A**). Along with the enrichment of gene signatures of inflammatory cells and pathways, we also found evidence of kidney damage in TGN and CGN mice with de-enrichment of the gene signature for podocytes (**Figure 2B**).

Relative gene expression results from the TI of NZM2328 kidneys showed a progressive pattern of inflammation and kidney cell loss. The TI regions of TGN kidneys were enriched for numerous immune and inflammatory signatures, including Th17 cells, PRRs, MHC class I and II, myeloid cells, and M1 and M2 Mϕs (**Figure 2C**). The TI of CGN mice exhibited enrichment of IFN stimulated genes, the cell cycle, and WNT signaling as well as decreases in gene signatures for mitochondria, amino acid metabolism, and oxidative phosphorylation (**Figure 2C**). We found further indicators of damage to the kidney tubules in CGN mice with decreases in kidney tubule cell gene signatures (**Figure 2D**) that correlated with significant increases in the expression of the kidney tubule damage-associated genes, *Havcr1* and *Lcn2* (**Figures 2E, F**). Overall, these results suggest that renal disease in female NZM2328 mice progresses from the glomerulus to the tubules and that inflammation established in the acute and transitional stages promotes a decrease in kidney cell signatures, indicative of cell damage.

### Lack of a robust inflammatory signature in glomeruli of NZM2328 male mice is associated with absence of progression to chronic renal disease

**Figure 3:**
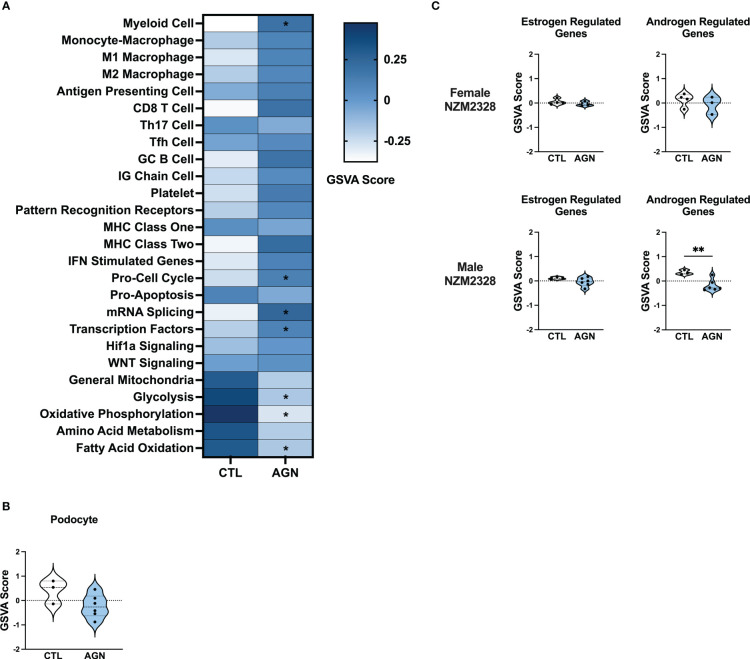
Male NZM2328 mice lack inflammatory signature enrichment associated with progression to chronic GN. **(A)** Heatmap of GSVA scores for enrichment of immune cell and pathway gene signatures in glomeruli of male CTL and AGN mice. Asterisks indicate significant comparisons with CTL mice. **(B)** GSVA enrichment of podocytes in cohorts shown in **(A)**. **(C)** GSVA enrichment of signatures for estrogen-regulated and androgen-regulated genes in glomeruli of female and male AGN mice. *p<0.05, **p<0.01.

To gain insight into the basis of the difference in gender-based resistance to chronic disease, we evaluated the transcriptomes of glomeruli from male NZM2328 mice with AGN at 10 months of age as compared to pre-disease, 8-9-week-old mice (**Figure 3**). Even though male mice were selected because they had evident immune complex deposition, male AGN mice, in contrast to the females, were not enriched for gene signatures indicative of a robust adaptive immune response or increased inflammation in the kidneys (**Figure 3A**). Instead, males exhibited enrichment for signatures of mRNA splicing and transcription factors and de-enrichment of metabolic pathways, including glycolysis, oxidative phosphorylation, and fatty acid oxidation. In addition, there was no difference in expression of kidney tissue signatures in male AGN mice as compared to normal controls (**Figure 3B**).

To assess the role of sex hormones in renal disease in NZM2328 mice, we developed signatures of estrogen- and androgen-regulated genes and compared their enrichment in female and male mice with AGN as compared to normal controls (**Figure 3C**). Female AGN mice exhibited no differences in enrichment of hormone-regulated gene signatures. However, in males, androgen-regulated genes were decreased in AGN mice. Furthermore, most androgen-regulated genes de-enriched in male mice were related to mitochondrial function and metabolic pathways, including *Akap1, Cox6b1, Iapp, Mrps6, Mybbp1a, Ndufa1, Phkg2, Prelid1, Sord*, and *Tmem86a.* This result suggested that decreased expression of male hormone response genes in AGN mice may contribute to resistance to disease progression by regulating metabolism and dampening inflammation.

### Inflammatory gene signatures in glomeruli of R27 mice differ from those in NZM2328 mice

We next examined the R27 congenic mouse strain as female R27 mice develop AGN with similar kidney pathology to NZM2328 mice, but do not progress further to severe proteinuria and ESRD. We compared gene expression profiles from glomeruli and TI of normal, CTL R27 mice (8-9 weeks) and R27 mice with AGN (12 months). R27 mice were selected for the presence of proteinuria and glomerular deposits of immunoglobulin detected by immunofluorescence. Examination of differentially expressed genes (DEGs) from glomeruli of NZM2328 and R27 mice relative to their respective CTLs (**Supplementary Table 3**, **Supplementary Figure 1A**) revealed significant overlaps with increased expression of APC, myeloid cell, Mϕ, and MHC signatures. In contrast, DEGs from NZM2328 but not R27 AGN mice showed overexpression of PRRs and IFN stimulated genes, suggesting a more severe inflammatory environment.

**Figure 4:**
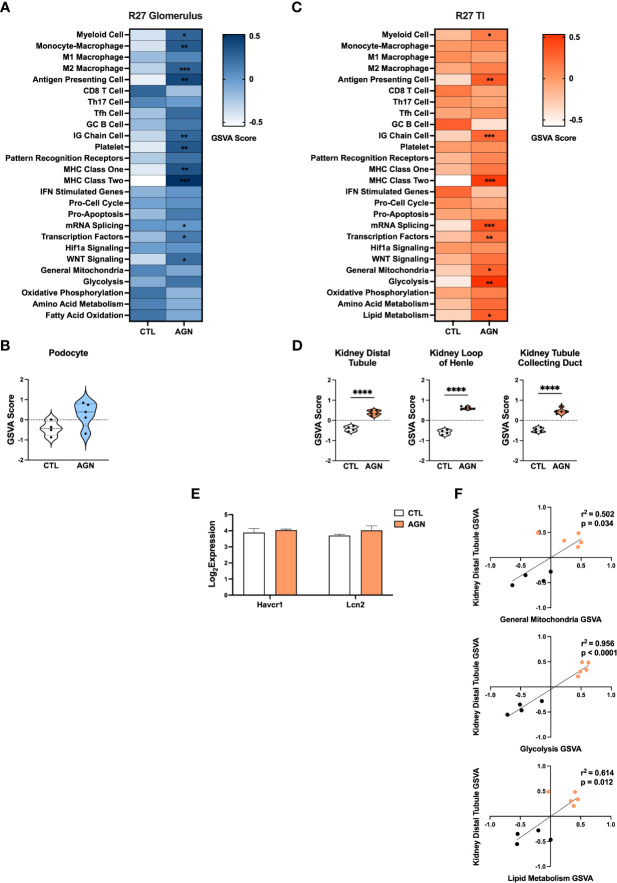
Molecular profiles of R27 mice differ from NZM2328 mice and indicate resistance to kidney tubule damage. **(A)** Heatmap of GSVA scores for enrichment of immune cell and pathway gene signatures in the glomeruli of R27 CTL and AGN mice. Asterisks indicate significant comparisons with CTL mice. **(B)** GSVA enrichment of podocytes in cohorts shown in A. **(C)** Heatmap of GSVA scores in the TI of cohorts shown in A. **(D)** GSVA enrichment of kidney tissue cell signatures in cohorts shown in C. **(E)** Log2 expression values of kidney tubule damage-associated genes for cohorts from C. **(F)** Linear regression between GSVA scores of kidney tubule cell and metabolic pathway gene signatures. *p < 0.05, **p < 0.01, ***p < 0.001, ****p < 0.0001.

GSVA analysis of R27 AGN glomeruli (**Figures 4A, C**) demonstrated enrichment of gene signatures indicative of inflammation, including APCs, IG Chains, Mϕs, and MHC class I and II (**Figure 4A**). Notably, analysis of Mϕ subsets revealed enrichment of anti-inflammatory M2, but not pro-inflammatory M1 Mϕs in R27 mice. No enrichment of IFN stimulated genes, PRRs, or Hif1a signaling between R27 control and AGN mice was detected. Moreover, no evidence of change in kidney cell-specific gene signatures was found in R27 AGN (**Figure 4B**).

### NZM2328.R27 mice exhibit resistance to kidney tubule damage

Next we compared DEGs expressed in the TI of R27 AGN mice relative to NZM2328 mice with AGN (**Supplementary Table 3**, **Supplementary Figure 1B**). DEGs from the TI of NZM2328 AGN mice were indicative of APCs, myeloid cells, Mϕs, and MHC class II, indicating the presence of some immune infiltrates but not to the same extent as the inflammation in the glomeruli at this early point in disease. In contrast, DEGs from R27 AGN mice were only indicative of APC and MHC class II and were not associated with other inflammatory signatures.

Similarly, GSVA of the TI of R27 AGN mice confirmed the enrichment of APCs, IG chains, and myeloid cell signatures, but not Mϕs, denoting a less extensive infiltration of immune/inflammatory cells in the R27 mice. (**Figure 4C**). Previous studies using the R27 strain found evidence that resistance to end organ damage might contribute to their decreased development of CGN (13). In support of this, we found that kidney tubule cell signatures were significantly increased in R27 AGN mice (**Figure 4D**), whereas the kidney damage-associated genes, *Havcr1* and *Lcn2*, were unchanged (**Figure 4E**). We also found that gene signatures related to mitochondria, glycolysis, and lipid metabolism were increased (**Figure 4C**) and were significantly correlated with the kidney tubule cell gene signature (**Figure 4F**), suggesting that robust mitochondrial function may contribute to the kidney tubule cell enrichment observed in R27 AGN mice.

### Kidney cell signatures enriched in NZM2328.R27 mice correlate with expression of chronic GN risk locus genes

**Figure 5:**
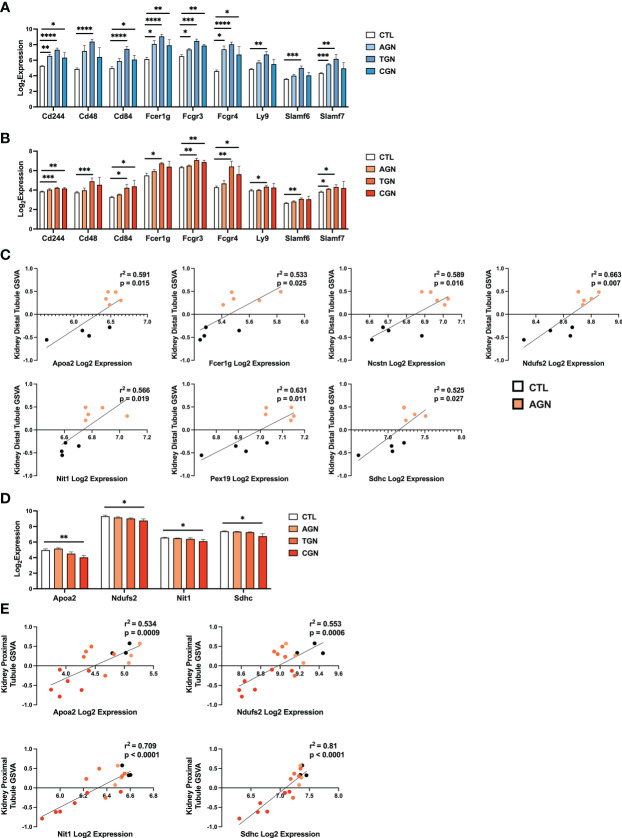
Expression of chronic risk locus genes is associated with disease severity and kidney tubule resistance in NZM2328 and R27 AGN mice. **(A, B)** Log_2_ expression values of immune receptor genes in the *Cgnz1* risk locus from glomeruli **(A)** and TI **(B)** of NZM2328 CTL, AGN, TGN, and CGN mice. **(C)** Linear regression between log_2_ expression of *Cgnz1* locus genes and GSVA scores of kidney tubule cells from the TI of R27 mice. All statistically significant correlations are shown. **(D)** Linear regression between log_2_ expression of *Cgnz1* locus genes from C and GSVA scores of kidney tubule cells from the TI of NZM2328 mice. All statistically significant correlations are shown. **(E)** Log_2_ expression values *Cgnz1* locus genes from C in the TI of NZM2328 CTL, AGN, TGN, and CGN mice. *p<0.05, **p<0.01, ***p<0.001, ****p<0.0001.

The risk for progression to CGN in NZM2328 mice was associated with a 1.34 Mb region of chromosome 1 (*Cgnz1*) containing 45 genes (13). We analyzed differential expression of these CGN susceptibility genes in glomeruli and TI of female NZM2328 AGN/TGN/CGN, and R27 AGN mice as compared to normal controls to determine their contribution to renal disease progression (**Figure 5**). In the glomerulus, we found that genes encoding receptors expressed on immune cells and associated with inflammation, including *Cd244, Fcer1g, Fcgr3, Fcgr4*, and *Slamf7*, were significantly increased in NZM2328 AGN, whereas none was overexpressed in R27 kidneys (**Figure 5A**; **Supplementary Figure 2**). Expression of these genes as well as additional immune-associated *Cgnz1* locus genes was further increased at the height of inflammatory cell and pathway gene signature enrichment at the TGN stage and either maintained or decreased at the CGN stage in glomeruli of NZM2328 mice.

In the TI, there were no significant differences in expression of *Cgnz1* locus genes in NZM2328 or R27 female AGN mice as compared to normal controls (**Supplementary Figure 3**). Furthermore, this result was consistent with the minimal inflammatory signature observed in AGN mice. However, at the TGN stage of NZM2328 mice, expression of immune-associated *Cgnz1* locus genes increased significantly over normal control mice, providing further evidence for the critical role of the CGN risk locus in progression to chronic disease (**Figure 5B**)

To investigate the relationship between CGN risk locus gene expression and kidney tubule cell enrichment in R27 female AGN mice, we carried out linear regression analysis (**Figure 5C**), and found that log_2_ expression values for 7 of the 45 genes composing the *Cgnz1* locus (*Apoa2, Fcer1g, Ncstn, Ndufs2, Nit1, Pex19, Sdhc*) were significantly correlated with GSVA scores for kidney distal tubule cells and thus could play a role in promoting resistance to kidney damage. Notably, we found that the proteins encoded by these genes were involved in mitochondrial respiration (*Ndufs2, Sdhc*), metabolite processing (*Apoa2, Ncstn, Nit1, Pex19*) and immune signaling (*Fcer1g*). Furthermore, log_2_ expression values for 4 of these genes (*Apoa2, Ndufs2, Nit1*, and *Sdhc*) were significantly correlated with kidney tubule cell GSVA scores and significantly decreased in the TI of NZM2328 CGN mice as compared to normal controls (**Figures 5D, E**).

To delve further into the functional pathways involving these kidney cell-associated genes, we identified upstream regulators (UPRs) using Ingenuity Pathway Analysis (IPA) (22) (**Supplementary Table 4**). Notable UPRs predicted to drive expression of the 7 *Cgnz1* genes correlated to kidney tubule cell enrichment included Rb1, Rictor, Wnt3a, Ctnnb1, and Hif1a and thus reflected the involvement of cell growth regulation, WNT signaling, and hypoxic stress response pathways in the kidneys of R27 AGN mice. These results suggest that the cellular functions associated with expression of some of the *Cgnz1* risk locus genes in R27 mice could contribute to robust mitochondrial function and promote resistance to kidney tissue damage in the context of acute nephritis.

### Gene co-expression network analysis identifies molecular profiles correlating with disease progression in NZM2328 mice

**Figure 6:**
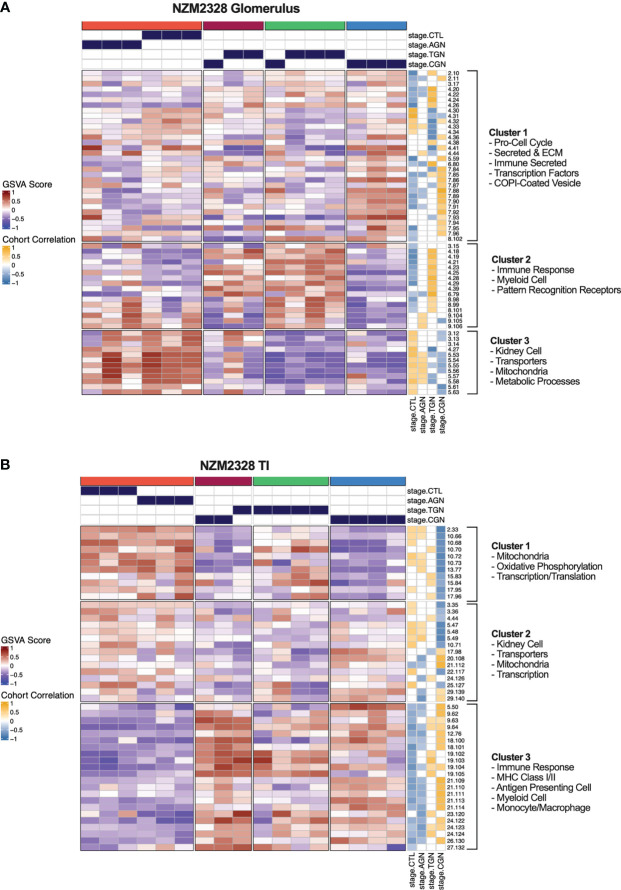
Unsupervised gene co-expression network analysis defines molecular profiles of NZM2328 mice correlated with disease severity. **(A, B)** K-means clustering (k=4) of NZM2328 CTL, AGN, TGN, and CGN mouse glomeruli **(A)** and TI **(B)** based on GSVA enrichment scores of MEGENA modules. The optimal number of module clusters was defined by the silhouette method and annotated by gene overlap with curated immunologic signatures and GO terms. Heatmap visualizations depict positive to negative GSVA scores on a red to blue gradient and positive to negative correlations between GSVA scores and disease classification on a gold to blue gradient.

As an orthogonal approach to identify molecular patterns reflective of disease stage in NZM2328 mice in an unsupervised manner, we generated a network of co-expressed gene modules using multiscale embedded gene co-expression network analysis (MEGENA) (23) and correlated individual gene modules with mouse GN stages (**Figure 6**). MEGENA of gene expression results from NZM2328 mice generated 60 co-expressed gene modules for the glomerulus and 48 modules for the TI that were divided into three megaclusters and annotated based on gene overlap with curated gene signatures as well as gene ontology (GO) terms (**Supplementary Table 5**). Overall, the MEGENA-derived gene modules were representative of the major cell types and processes we had previously associated with GN using curated gene signatures, including inflammatory myeloid cells, kidney tissue cells, and metabolic processes. Furthermore, k-means clustering based on the MEGENA modules successfully separated mice into cohorts based on disease severity. In the glomerulus (**Figure 6A**), the coral cluster of CTL and AGN mice was positively correlated with gene modules associated with kidney cells and metabolic processes, and negatively correlated with gene modules related to the immune/inflammatory response. Two clusters (maroon and green) contained a combination of TGN and CGN mice and were positively correlated with immune response modules and negatively correlated with kidney cell and metabolism modules. The final cluster of CGN mice (blue) had a negative correlation with immune response and kidney/metabolic modules but retained a high positive correlation with secreted immune factors. MEGENA results and correlations with disease stage in the TI (**Figure 6B**) were similar to the glomerulus, but the resulting gene modules were more heavily skewed toward mitochondrial metabolism and the blue cluster of CGN mice was still positively correlated with the immune response-associated modules. In summary, this unsupervised approach employing co-expressed gene modules yielded results that closely resemble our previously identified molecular profiles of disease progression in NZM2328 mice.

### Identification of gene signatures characterizing GN stages in NZM2328 mice

**Figure 7:**
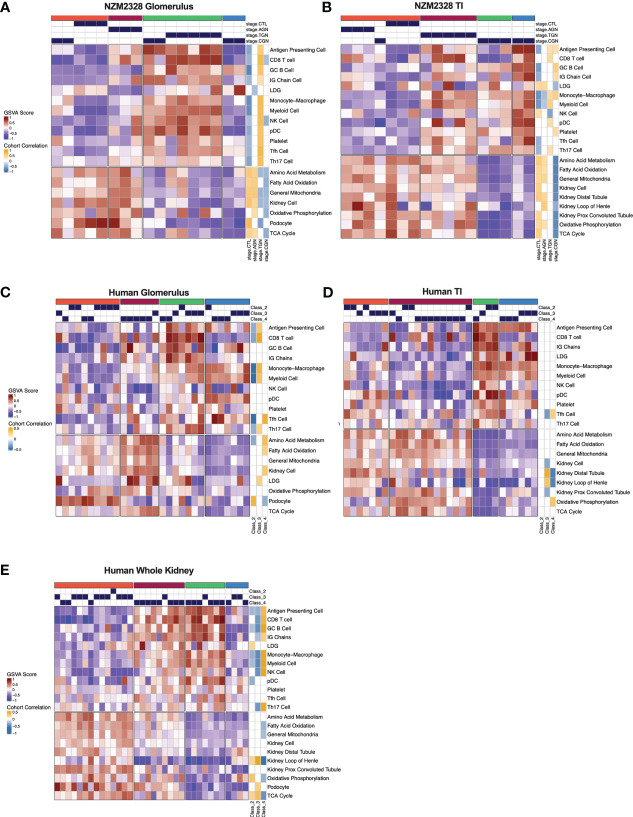
Gene signature-based clustering of GN stages in NZM2328 mice translates to human LN patients. **(A, B)** K-means clustering (k=4) of NZM2328 CTL, AGN, TGN, and CGN mouse glomeruli **(A)** and TI **(B)** based on GSVA enrichment scores of selected immune cell, kidney cell, and metabolic pathway gene sets. **(C-E)** K-means clustering (k=4) of microdissected glomeruli **(C)**, TI **(D)**, and whole kidney **(E)** from human LN patients based on GSVA score from human orthologs of the mouse gene sets used in **(A, B)**. Heatmap visualizations depict positive to negative GSVA scores on a red to blue gradient and positive to negative correlations between GSVA scores and disease classification on a gold to blue gradient.

We next sought to assemble a panel of curated gene signatures that would characterize the inflammatory environment in different stages of murine GN and determine whether similar immune profiles could be identified in human LN kidneys. To accomplish this, a core set of 22 GSVA gene signatures was selected based on significant enrichment in AGN, TGN, or CGN NZM2328 mice (**Supplementary Table 2**; **Figures 2A, C**). GSVA scores were then used as input for k-means clustering to form 4 clusters of mice from the glomerulus and TI gene expression datasets (**Figures 7A, B**). In the glomerulus, AGN mice in the maroon cluster were characterized by slightly increased enrichment of inflammatory immune cells compared to CTL mice but retained enrichment of kidney tissue cell and metabolism gene signatures (**Figure 7A**). TGN mice in the green cluster exhibited the highest enrichment of all inflammatory gene signatures accompanied by a decrease in metabolic and kidney cell signatures. CGN mice were divided among multiple clusters and thus reflected heterogeneity in immune profiles among mice at this stage of disease. Two CGN mice were placed in the coral cluster with CTL mice reflecting waning inflammation and retention of metabolic and kidney cell signatures. Another group of CGN mice with continued evidence of inflammatory gene signatures were found in the green cluster with TGN mice. Finally, the blue cluster of CGN mice exhibited a relative de-enrichment of immune cells, kidney cells, and metabolic pathways indicative of a post-inflammatory state with evidence of end organ damage.

Gene expression-based clustering of the TI yielded similar results as the glomerulus with increasing inflammation and decreasing metabolism and kidney tubule cell gene signatures marking progression in disease severity (**Figure 7B**). However, in the TI, the AGN mice clustered with CTLs and more of the CGN mice appeared to retain immune cell enrichment, reflecting persistent immune cell infiltration.

### Validation of NZM2328 gene expression patterns in an unrelated dataset

To validate the findings in NZM2328 mice in another lupus-prone strain, we applied the same approach to analysis of publicly available gene expression data from whole kidney tissue of the IFNα-accelerated NZB/W model (IFNα-NZB, GSE86423). Notably, the 22 curated gene expression signatures used to separate disease stages in NZM2328 mice, followed a similar enrichment pattern over a 9-week time course in IFNα-NZB mice indicating that this result was not unique to the gene expression dataset we generated from the NZM2328 strain (**Supplementary Figure 4**).

### Gene signatures characterizing GN stages in NZM2328 mice identify analogous subsets of human LN patients

To determine whether immune profiles of NZM2328 mice with different stages of GN would translate to human lupus patients, we analyzed a publicly available gene expression dataset of microdissected glomeruli and TI from kidneys of patients with International Society of Nephrology (ISN) class II-IV LN as determined by histological classification (GSE32591) (27). We carried out GSVA using human orthologs of the 22 curated mouse gene signatures and identified 4 molecular endotypes by k-means clustering based on the pattern of enriched gene signatures in each individual patient (**Supplementary Table 1**; **Figures 7C–E**). GSVA results of glomeruli and TI from kidneys of LN patients formed 4 patient clusters that exhibited similar gene set enrichment profiles to the nephritic kidneys of NZM2328 mice (**Figures 7C, D**). In both the glomerulus and TI, we observed a clear progression with increased enrichment of inflammatory cells corresponding with de-enrichment of kidney tissue cells as well as metabolic pathway signatures. In addition, the cohort correlations between LN classification and gene signature enrichment revealed increased correlations with pro-inflammatory cells in proliferative nephritis, particularly in the glomerulus, and corresponding negative correlations with kidney tissue cell signatures. However, whereas in the mouse kidneys we observed a post-inflammatory cluster of CGN stage mice, human LN samples with the greatest de-enrichment in metabolic and kidney cell signatures also retained a relatively high enrichment of immune/inflammatory cell signatures.

To confirm these results, we generated and analyzed a second gene expression dataset from whole kidneys of human LN patients in a similar manner (**Figure 7E**). Notably, gene signature enrichment profiles of each human whole kidney subset more closely resembled clusters from mouse GN, including a cluster of samples that exhibited both de-enrichment of inflammatory signatures and metabolic signatures. GSVA of human whole kidney gene expression using the unsupervised MEGENA modules generated for NZM2328 mouse kidneys (**Figure 6**) also yielded similar patterns of gene expression enrichment across LN patient clusters (**Supplementary Figure 5**). As an additional approach to establish similarities between mouse and human kidney gene expression profiles, we carried out MEGENA using the human LN whole kidney dataset. Then, MEGENA modules generated for the NZM2328 mouse (**Figure 6**) were used as a reference to determine the preservation of gene module assignment between the mouse and human kidney gene co-expression networks (**Supplementary Table 5**). The results indicated that 22 MEGENA modules from the mouse glomerulus and 31 MEGENA modules from the mouse TI had a significant module preservation score (z-score > 2) with human kidney modules indicating a high degree of overlap in their gene expression profiles. Overall, these results demonstrate that gene expression analysis can be used to classify stages of GN in lupus-prone mice and that mouse kidney endotypes can be translated to human LN patients.

## Discussion

The challenge of classifying disease pathology in heterogeneous presentations of LN has highlighted the need for a better understanding of disease progression in the kidneys of lupus patients and the risk factors for ESRD. To begin to address this, we utilized gene expression analysis to characterize stages of autoimmune inflammation leading up to the development of chronic disease in an established murine model of human GN. Mice were classified in disease stages by histological comparison, matching mice by level of disease pathology and amount of IC deposition. This analysis revealed distinct immune profiles for acute disease, after initial IC deposition in the kidney glomerulus, transitional disease in which inflammatory cell and pathway enrichment is at its peak, and chronic disease in which the accumulated insults result in irreversible damage to the kidney tissue.

We found evidence of selective immune cell infiltration in glomeruli of AGN mice, including enrichment for Monocyte/Mϕ, APC, MHC Class II, and Tfh cell gene signatures. This result reflects a limited set of immune/inflammatory cells present in the tissues at the initiation of AGN and likely reflects the cellular response to IC deposition (1, 12, 28). Enrichment of apoptosis and PRR gene signatures at the AGN stage may reflect the widening innate immune response triggered by early DAMP release from the kidney tissue. At the AGN stage, glomeruli of NZM2328 mice were also enriched for CD8 T cells, which studies have found to be elevated in both human and mouse GN and have been linked with disease severity (29–31). However, it is possible that in the context of AGN, these CD8 T cells act in a more regulatory rather than effector cell capacity, as previously suggested (32).

Our classification of the progression of GN in NZM2328 mice uncovered a newly recognized transitional stage during which we observed the greatest level of immune activity. As NZM2328 mice progressed to TGN, we observed a striking increase in innate immune response pathways and evidence of significant myeloid cell infiltration in the kidney tissue. Previous molecular studies of LN report a robust IFN response as the key feature distinguishing kidneys of lupus patients from healthy individuals and the TGN stage is when we first observed significant enrichment of an IFN signature in glomeruli of diseased mice (33–35). In line with this result, we also found significant enrichment of Mϕ populations in TGN mice and, in particular, those with a pro-inflammatory, M1 rather than an alternatively activated, M2 gene signature. Mϕs with both an M1 and an M2 phenotype have been described in mouse models of LN and associated with disease pathogenesis (36–39). However, despite the production of anti-inflammatory molecules by M2 Mϕs, the amplification of inflammatory cytokine production by immune and kidney tissue cells was found to overwhelm any regulatory response and promote disease progression. Kidney-infiltrating Mϕs are also important mediators of damage to the kidney tissue and we found that increases in Mϕ signatures in TGN mice were accompanied by decreases in kidney cell signatures and, in particular, podocytes. Podocytes are frequent targets of immune infiltration in the glomerulus and podocyte injury has been associated with proteinuria in lupus patients and is regarded as a precursor to end organ renal damage (40–42).

It has been reported that the low oxygen tension environment in the kidney becomes more hypoxic in LN, correlates with disease severity, and is associated with mitochondrial dysfunction in lupus mouse models (43, 44). In addition, several studies supporting the “chronic hypoxia hypothesis” have identified hypoxia-induced damage in the TI as the final critical pathway leading to ESRD in human patients (3, 5, 45). Our results align with these studies as we observed enrichment of the hypoxia response pathway through Hif1a in the glomeruli of TGN mice. Furthermore, heightened severity of disease pathology in CGN mice was accompanied by evidence of further damage to the kidney tissue, as well as a loss of mitochondrial and metabolic gene signatures suggestive of mitochondrial dysfunction. Therefore, our results support previous assertions that targeting the hypoxia response and mitochondrial dysfunction may be beneficial in the treatment of lupus patients (44, 46).

Glomeruli serve as the first connection points of kidney nephrons with the vasculature before disease progresses downstream to the kidney tubules such that kidney tubule damage is regarded as a diagnostic marker for progression to ESRD (7, 47). In line with this, enrichment of inflammatory cell and pathway gene signatures was delayed in the TI as compared to glomeruli of nephritic mice and resulted in de-enrichment of kidney tubule cell gene signatures in the TI of CGN mice. We also found that the expression of kidney-damage associated genes *Havcr1* and *Lcn2* (48–51) was significantly elevated in the TI of CGN mice. In addition, de-enrichment of metabolic gene signatures indicative of mitochondrial dysfunction was more prevalent in the TI of CGN mice suggesting that mitochondrial stress contributed to kidney tubule damage in late-stage disease.

Here, we also examined the mechanism(s) of resistance to chronic disease based on differences in gender and genetic background of lupus-prone NZM2328 mice. The increased prevalence of SLE in females over males in both human lupus patients and certain lupus mouse models implicates sex hormones in the pathogenesis of LN (52–55). Our analysis by both histology and gene expression-based approaches confirmed that male NZM2328 lupus-prone mice develop a milder form of AGN than female mice that does not progress to CGN (14). In addition, critical metabolic signatures, including glycolysis and oxidative phosphorylation, were decreased in male AGN mice suggestive of a dampened inflammatory response. Analysis of sex hormone-regulated gene signatures in the kidney did not indicate a difference in the estrogen response of female or male mice, which has been associated with lupus pathogenesis in both humans and mouse models (56–59). However, in many cases, the effects of estrogen regulation have been on immune cell populations and, therefore, we cannot discount an influence of estrogen regulation on circulating immune cells outside of the kidney tissue. In contrast to estrogens, androgens have been implicated in immunosuppression with decreased levels found in autoimmunity (60, 61). In line with this, androgen-regulated genes were de-enriched in male NZM2328 AGN mice and the genes contributing to this decrease were involved in cellular metabolism, suggesting a mechanism of androgen regulated immunosuppression through targeting metabolic pathways that is decreased in NZM nephritic mice.

We investigated the genetic-based resistance to chronic disease using female mice of the congenic strain, NZM2328.R27 (13). Interestingly, glomeruli of R27 mice exhibited evidence of anti-inflammatory, M2 Mϕ infiltration with no enrichment of the pro-inflammatory, M1, gene signature observed in the base strain. This result suggests that the altered nature of the inflammatory response in R27 AGN mice contributes to end organ resistance to disease. Furthermore, the TI of R27 AGN mice exhibited enrichment of gene signatures indicating a resistance to damaging pathologic processes stemming from inflamed glomeruli including increased kidney tubule cell signatures in conjunction with increased mitochondrial and metabolic gene signatures.

Since the R27 strain was derived by replacing the chronic disease risk locus, *Cgnz1*, of NZM2328, we examined the potential contribution of the 45 genes within this locus to resistance to CGN. We uncovered several pro-inflammatory genes with elevated expression in NZM2328 female mice, that would promote the activation of pathogenic immune populations such as M1 Mϕs and have been implicated in GN (62, 63). Furthermore, 7 risk locus genes that significantly correlated with kidney tubule cell signature enrichment in R27 AGN mice were involved in cell growth, metabolism, and WNT signaling. Involvement in boosting mitochondrial function could counteract the risk of mitochondrial stress and loss of function that were present in late-stage NZM2328 female mice. In addition, WNT signaling has been shown to have a positive role in resolving acute kidney injury, whereas it may promote maladaptive responses during chronic disease (64).

We have identified multiple mechanisms by which lupus-prone mice acquire resistance to chronic nephritis with implications for identifying risk factors for ESRD in human lupus patients. Interestingly, these mechanisms appear to be independent of the amount of IC deposition as all AGN mice (NZM2328 female, NZM2328 male, and R27 female) were matched by the level of pathology before monitoring disease progression. Resistance to chronic disease in male NZM2328 mice may have occurred at the initial point of IC deposition in the glomerulus, which failed to elicit a potent inflammatory response, possibly related to androgen-dependent suppression of energy-producing metabolic pathways. Resistance to chronic disease in R27 mice was associated with an altered composition of immune cells in the glomerulus that resulted in a lack of immune pathology downstream in the tubules. Moreover, the tubules in the R27 mice appear to be resistant to damage, as manifested by enhanced metabolic signatures. The resistance of tubules to damage related to immune activity in the glomerulus and/or hypoxia could play a pivotal role in preventing the typical inflammatory infiltrate in the TI of CGN, Thus, the absence of tubular dysfunction may have limited the inflammatory infiltrate in the TI and ultimately prevented additional damage to the kidney tissue.

Using a gene expression-based clustering approach, we have identified a core set of curated gene signatures able to classify disease stages of murine GN into molecular endotypes that effectively translate to human LN patients. Notably, human orthologs of the murine GN gene signatures identified a similar pattern in two independent cohorts of human LN patients consisting of increased enrichment of inflammatory cells and corresponding de-enrichment of metabolic pathways and kidney tissue cells associated with more advanced stages of kidney pathology. In current practice, the severity of LN pathogenesis is determined by histological classification, which is used to drive therapeutic decisions and assess the potential for terminal kidney damage (27, 65, 66). We found only modest correlation between ISN histological classification of renal pathology in human LN patients and molecular classification by gene expression profiling and the gene signature correlations that were identified were inconsistent across patients with the same ISN class and between datasets. This result emphasizes the subjectivity of histological assessment of renal pathology, and suggests that molecular classification may be a more robust and reproducible approach to classification of human LN.

An orthogonal, unsupervised approach to generate co-expressed gene modules (MEGENA) also identified similar molecular patterns that effectively classified mouse GN stages, human LN patients, and were highly conserved between species. This unsupervised approach supplies further validation for gene expressed-based profiles derived from curated gene signatures as well as the utility of lupus mice to recapitulate human LN at the molecular level. In summary, this work provides a comprehensive examination of the immune processes involved in progression of murine GN to chronic disease resulting in renal failure. In addition, this work presents a foundation for improved classification of LN based on molecular endotypes and illustrates the applicability of murine models to better understand the stages of human disease.

## Data availability statement

The murine microarray dataset generated for the current study is available from NCBI’s GEO database under accession GSE206806. The human microarray dataset generated for the current study is available from ArrayExpress under accession E-MTAB-12257. The publicly available murine and human microarray datasets analyzed in the current study can be found under GEO accessions GSE86423 and GSE32591, respectively.

## Ethics statement

The studies involving human participants were reviewed and approved by Shanghai Institute of Rheumatology. The patients/participants provided their written informed consent to participate in this study. The animal study was reviewed and approved by the University of Virginia Animal Care and Use Committee.

## Author contributions

Conceptualization: AD, HW, SF, and PL. Methodology: AD, HW, SF, and PL. Software: PB. Formal analysis: AD, HW, PB, KK, and RR. Data Curation: HW, SF, NS, PB, KK, and RR. Writing – original draft: AD. Writing – review & editing: AD, HW, KK, NS, RR, and PL. Visualization: AD and HW. Supervision: NS, SF, AG, and PL. Project administration: NS, SF, AG, and PL. Funding acquisition: SF, AG, and PL. All authors contributed to the article and approved the submitted version.
