# Supplementary material for: Molecular mechanisms governing the progression of nephritis in lupus prone mice and human lupus patients
Source: Front Immunol. 2023 Mar 1;14:1147526. doi: 10.3389/fimmu.2023.1147526 (PMC10016352; doi:10.3389/fimmu.2023.1147526)
Supplement: Supplementary file 1 [file DataSheet_1.pdf]

frontiers

Supplementary Figures

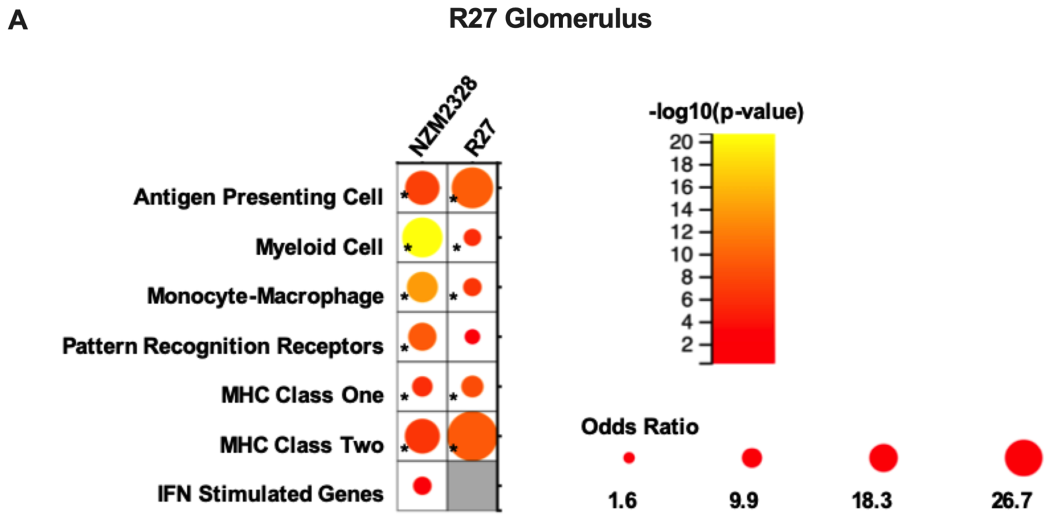

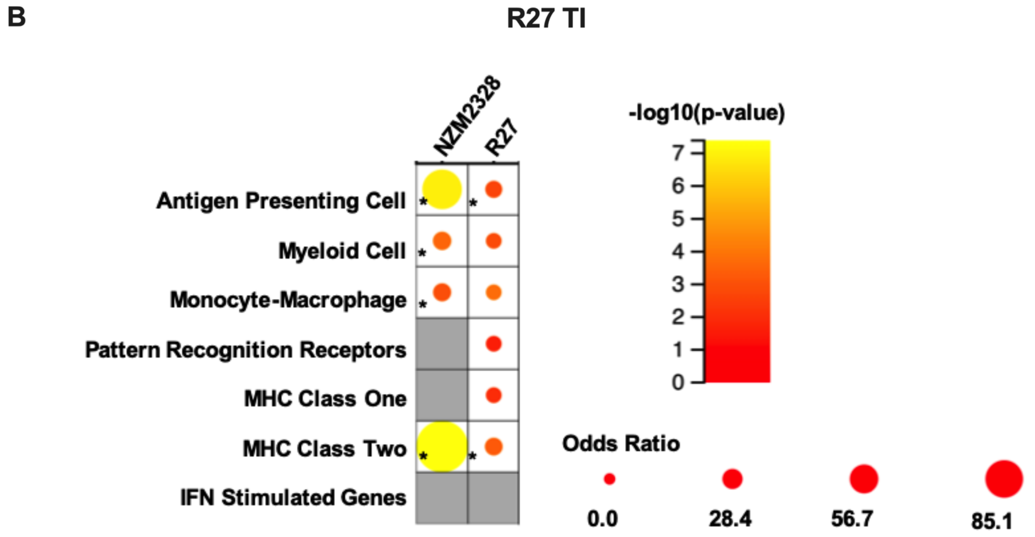

Supplementary Figure 1. Differential gene expression analysis of NZM2328 and R27 AGN mice. (A&B) Bubbleplot depicting the overlap of DEGs up-regulated in the glomeruli (**A**) and TI (**B**) of NZM2328 and R27 AGN mice with immunologic gene signatures. Bubble size indicates odds ratio and color indicates p-value of the comparison with CTL mice. Asterisks indicate statistically significant comparisons.

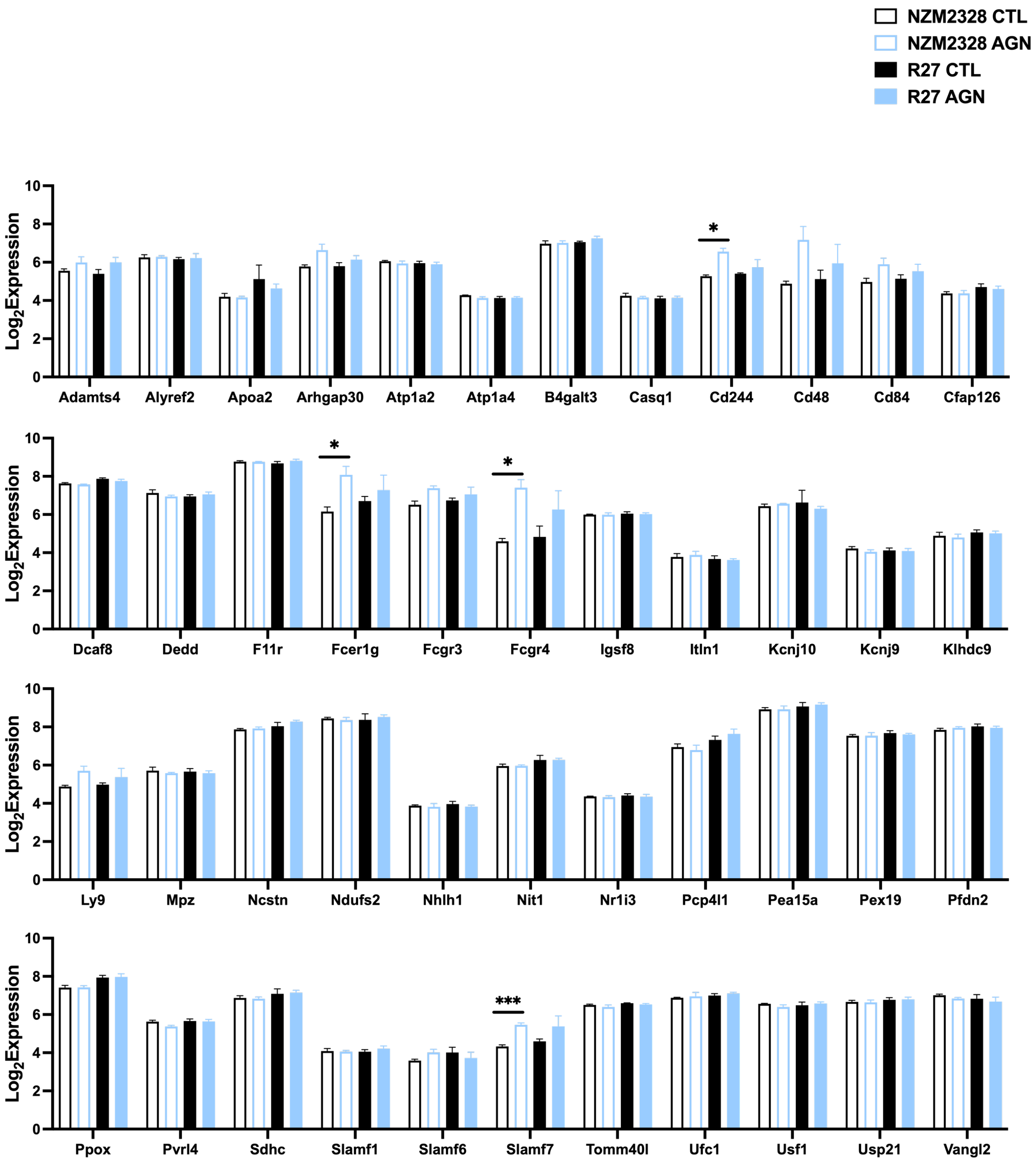

Supplementary Figure 2. Expression of *Cgnz1* locus genes in the glomeruli of NZM2328 and R27 AGN mice. Log_2 expression values of genes in the *Cgnz1* risk locus from the glomeruli of NZM2328 and R27 CTL and AGN mice. Statistical significance was evaluated separately for NZM2328 CTL vs AGN and R27 CTL vs AGN comparisons.

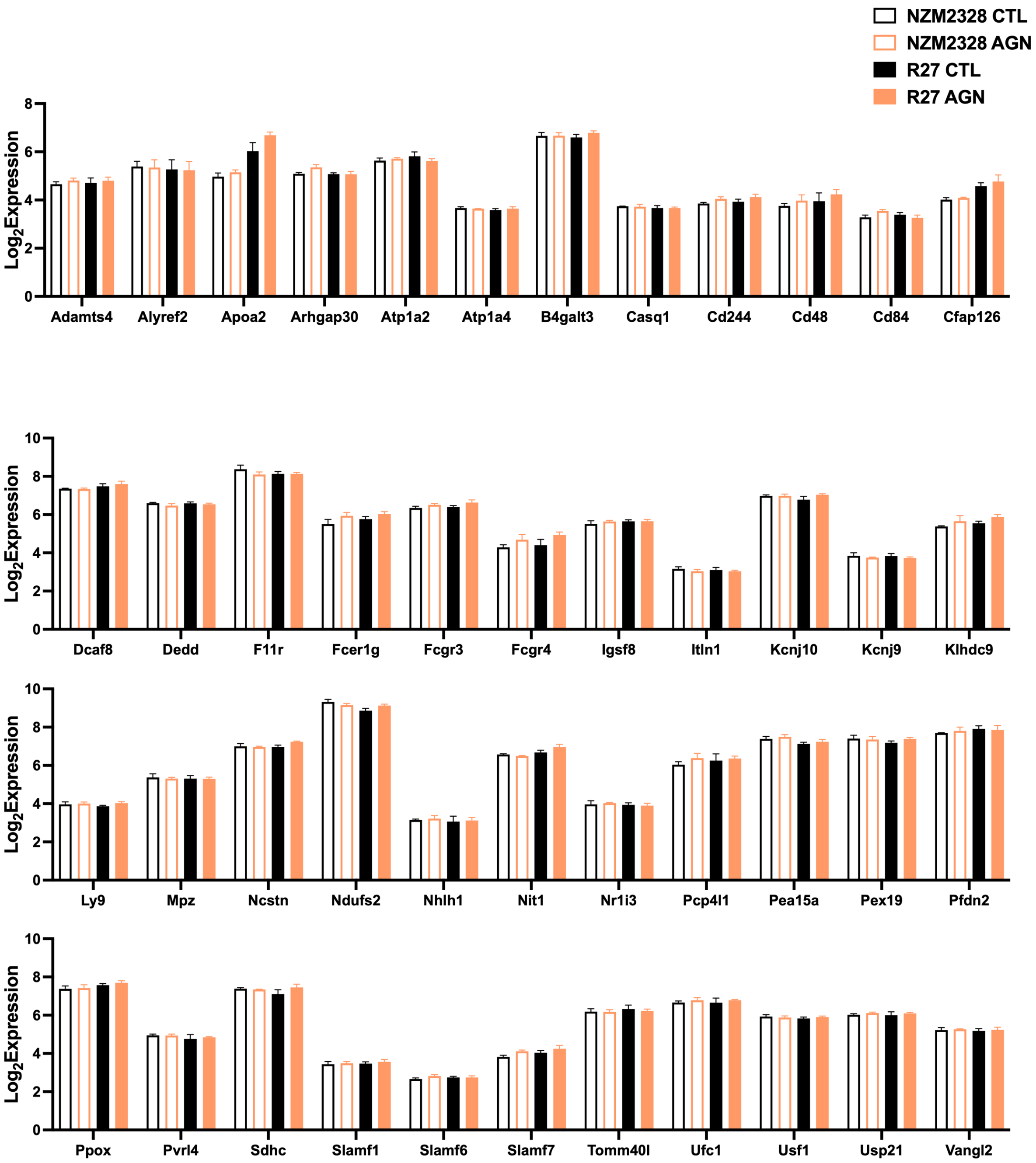

Supplementary Figure 3. Expression of *Cgnz1* locus genes in the TI of NZM2328 and R27 AGN mice. Log_2 expression values of genes in the *Cgnz1* risk locus from the TI of NZM2328 and R27 CTL and AGN mice. Statistical significance was evaluated separately for NZM2328 CTL vs AGN and R27 CTL vs AGN comparisons.

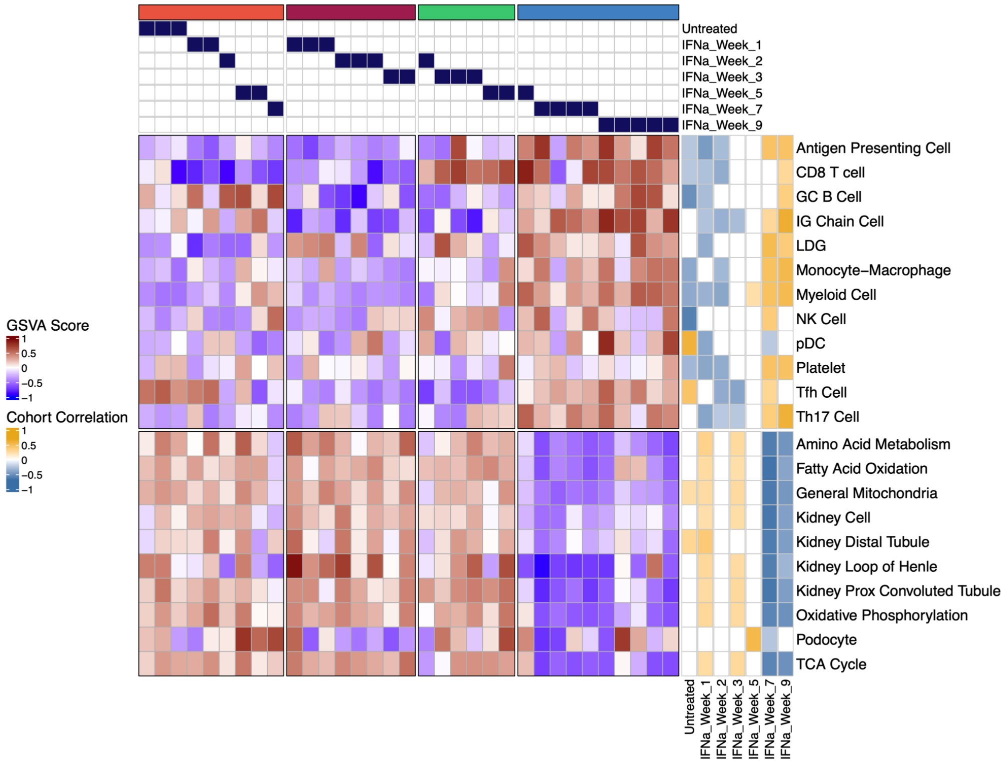

Supplementary Figure 4. Gene signature-based clustering of IFNα-NZB mouse kidneys. K-means clustering (k=3) of IFNα-NZB mice over time after IFNα treatment based on GSVA enrichment scores of selected immune cell, kidney cell, and metabolic pathway gene sets. Heatmap visualizations depict positive to negative GSVA scores on a red to blue gradient and positive to negative correlations between GSVA scores and disease classification on a gold to blue gradient.

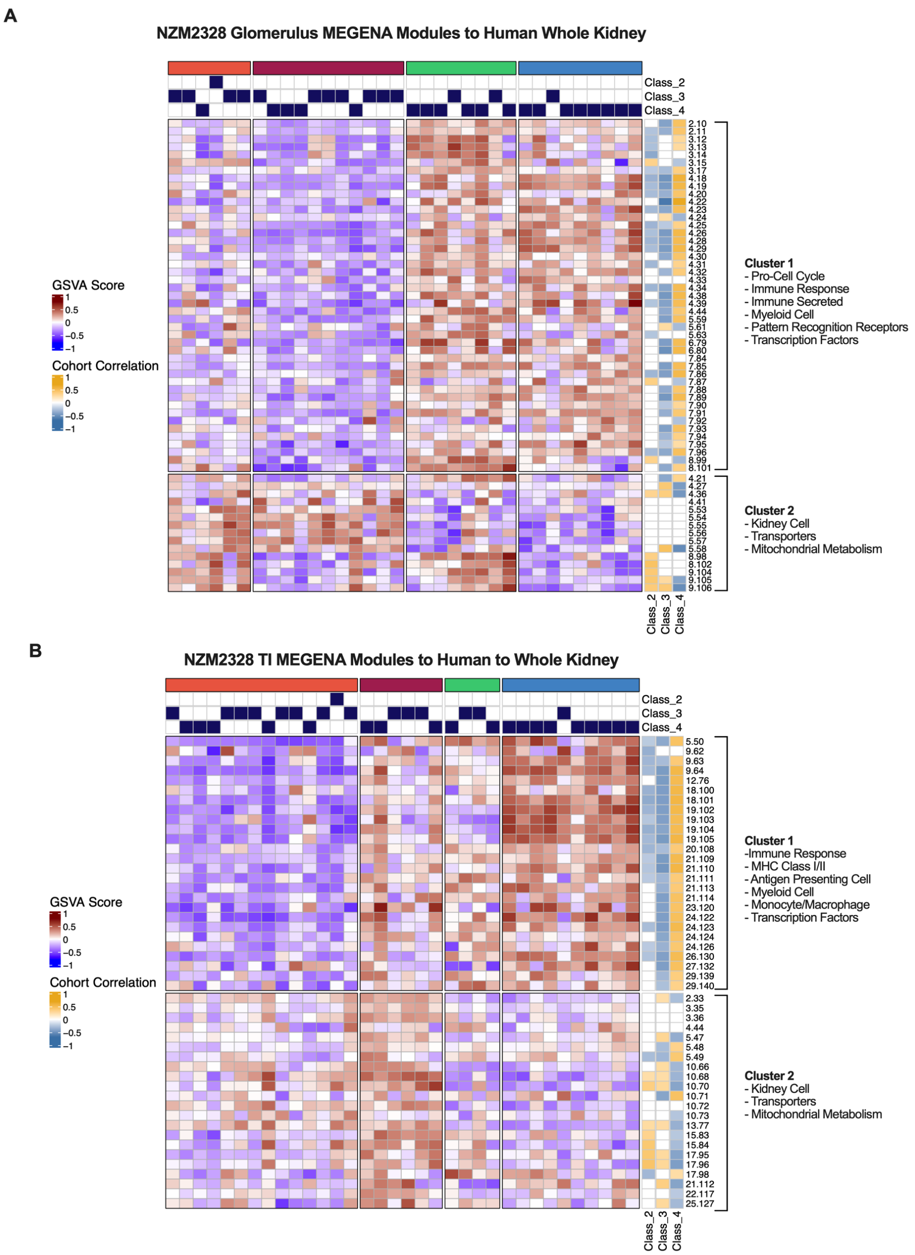

Supplementary Figure 5. NZM2328 mouse MEGENA module-based clustering of human LN kidneys. K-means clustering (k=4) of whole kidney samples from human LN patients based on GSVA scores from human orthologs of the MEGENA modules from NZM2328 mouse microdissected glomeruli (**A**) and TI (**B**) used in Figure 8. The optimal number of module clusters was defined by the silhouette method and annotated by gene overlap with curated immunologic signatures and GO terms. Heatmap visualizations depict positive to negative GSVA scores on a red to blue gradient and positive to negative correlations between GSVA scores and disease classification on a gold to blue gradient.
